# Changes in circulating lipids level over time after acquiring HCV infection: results from ERCHIVES

**DOI:** 10.1186/s12879-015-1268-2

**Published:** 2015-11-11

**Authors:** Adeel A. Butt, Peng Yan, Tracey G. Simon, Raymond T. Chung, Abdul-Badi Abou-Samra, for the ERCHIVES study team

**Affiliations:** VA Pittsburgh Healthcare System, 3601 Fifth Avenue, Suite 3A, Pittsburgh, PA 15213 USA; University of Pittsburgh School of Medicine, Pittsburgh, PA USA; Hamad Healthcare Quality Institute, Doha, Qatar; Hamad Medical Corporation, Doha, Qatar; Massachusetts General Hospital, Boston, MA USA

**Keywords:** HCV, Lipid, Cholesterol, LDL, ERCHIVES, Seroconversion

## Abstract

**Background:**

Changes in lipid levels over time after acquiring HCV infection, and how they differ from HCV-uninfected persons are unknown.

**Methods:**

We used ERCHIVES to identify those with a known HCV seroconversion window and persistently negative controls. We excluded subjects with HIV and hepatitis B and those who received lipid lowering agents. Total Cholesterol (TC), low-density lipoproteins (LDL), high-density lipoproteins (HDL), triglycerides (TG) and non-HDL cholesterol were retrieved at yearly intervals and plotted over time.

**Results:**

Among 1,270 HCV+ and 5,070 HCV- subjects, median age [IQR] was 47[37,53] for HCV+ and 52[47,57] for the HCV- group; 69 % were White and 91 % were males in each group. Mean BMI [SD] was 26.94[6.73] in the HCV+ and 28.15 [5.98] in the HCV- group (*P < 0.001)*. Over a 10-year follow-up period among HCV+ persons, TC decreased by (mean (SD) mg/dL) 12.06(36.95), LDL by 9.22(31.44), TG by 13.58(87.01) and non-HDL-C by 12.55(35.14). Among HCV- persons, TC cholesterol decreased by 4.15(31.21), LDL by 4.16(26.51); TG by 4.42(82.34) and non-HDL-C by 5.78(30.17).

**Conclusions:**

After HCV acquisition, TC, LDL, TG and non-HDL-C progressively decline over time independent of BMI and liver fibrosis. Consequences of lipid changes and the need and optimal timing of lipid lowering therapy in HCV+ persons require further study.

**Electronic supplementary material:**

The online version of this article (doi:10.1186/s12879-015-1268-2) contains supplementary material, which is available to authorized users.

## Background

Hepatitis C virus (HCV) infection has been associated with alterations in lipid levels. Persons with HCV infection have significantly lower total cholesterol (TC), low density lipoprotein (LDL) and triglyceride (TG) levels compared with demographically similar HCV uninfected controls. In a previous study of 82,083 HCV infected and 89,582 HCV uninfected controls, TC was 32 mg/dL lower, LDL was 18 mg/dL lower and TG were 25 mg/dL lower among HCV infected persons. [1] Other studies have shown similar associations.[2–4] Some previous reports offer a cross-sectional comparison at baseline for those particular studies and do not account for duration of HCV infection or changes in lipid profile over time. Others have reported on changes in lipid profiles after treatment for HCV.[5–8] To our knowledge, no studies have reported the changes in lipid profile after HCV acquisition and how they compare with demographically similar HCV uninfected persons.

It is currently unknown whether the changes in lipid profile among HCV infected persons are present even at time of HCV acquisition thus reflecting differences in demographic, behavioral and anthropometric parameters before infection or whether HCV infection itself triggers changes in the lipid profile. Temporal changes in lipid profile over time after HCV acquisition are also unknown. We used a previously described cohort with a known seroconversion timeframe within the Electronically Retrieved Cohort of HCV Infected Veterans (ERCHIVES) [9] to determine the changes in lipid profile over time among HCV infected persons after seroconversion and comparable HCV uninfected controls.

## Methods

### Study population

Construction of ERCHIVES has been described in several previous publications.[1, 10–18] Briefly, ERCHIVES contains all HCV infected persons identified via a positive HCV antibody test within the Veterans Affairs Healthcare System (VA) nationally between 2002–2013. Controls are age (5-year blocks), race and gender matched (1:1) with a negative HCV antibody test in the same year as the positive test for HCV infected group. Demographic, clinical, laboratory, pharmacy and mortality data are extracted from various national VA repositories and merged using scrambled social security numbers accordingly to a well-established algorithm.

For the current study, we identified persons within ERCHIVES with a first negative HCV antibody test followed by a positive HCV antibody test and no subsequent negative HCV antibody test. We excluded persons with HIV infection and a positive hepatitis B surface antigen (HBsAg). We also excluded persons with no lipid profile measurement at baseline and no measurement > 24 months after baseline. We further excluded persons with missing or undetectable HCV RNA and baseline and those who were prescribed any lipid lowering agents for >28 days. Persons who received HCV treatment were censored at the time of treatment initiation. For each person finally identified, we identified up to four controls, matched on age (5-year blocks), race and gender, who had at least one negative HCV antibody test within 12 months of the first positive HCV antibody test among the seroconversion group. Controls were further required to have at least one more negative HCV antibody test to account for the testing bias for the seroconversion group, and no positive HCV antibody test ever after. Those with HIV infection, positive hepatitis B surface antigen, missing lipid profiles and who received lipid lowering agents were excluded similar to the seroconversion group.

### Measurements

Lipid profiles were obtained during the course of routine clinical care. Since these values were obtained from electronic medical records, the proportion of persons who were fasting at time of testing is not known. Baseline value was taken to be the average of two most recent values prior to baseline. Lipid profiles were subsequently retrieved on yearly basis, using the values closest to the yearly time points. Non-HDL cholesterol (non-HDL-C) was calculated by subtracting HDL cholesterol from TC. Comorbidities were defined using lab values and/or disease codes using International Classification of Diseases, Ninth Revision, Clinical Modification (ICD-9 CM) as previously described.[1, 9] Use of lipid lowering agents was defined as prescription of any drug approved for the treatment of elevated lipid levels for >28 days. Body mass index (BMI) was calculated as weight divided by the square of height (kg/m^2^) and categorized into underweight (BMI  < 18.5), normal weight (BMI 18.5–24.9), overweight (BMI 25.0–29.9) and obese (BMI ≥ 30.0). Degree of liver fibrosis was measured by using FIB-4 score, calculated as follows:

$$ \mathrm{FIB}\hbox{-} 4 = \mathrm{age}\ \left[\mathrm{years}\right] \times \mathrm{A}\mathrm{S}\mathrm{T}\ \left[\mathrm{IU}/\mathrm{L}\right]/\mathrm{platelet}\ \mathrm{count}\ \left[\mathrm{platelets} \times {10}^9/\mathrm{L}\right] \times \left({\mathrm{ALT}}^{1/2}\left[\mathrm{IU}/\mathrm{L}\right]\right) $$

### Analysis

Baseline demographic and clinical characteristics and lipid profile were compared between HCV infected and uninfected persons using chi-square for dichotomous and *t*-test for continuous variables. We plotted changes in each of the lipid fractions, TC, LDL, HDL, TG and non-HDL-C, over time for HCV infected and uninfected persons. To understand the changes in lipid profile, we also tabulated and plotted each lipid fraction over time by BMI category (at baseline) and HCV genotype.

We performed additional analysis when plotting by HCV status, adjusting for severity of liver fibrosis using time-updated FIB-4 scores. A *p*-value of <0.05 was considered significant where comparisons were made. We used SAS® (SAS Institute Inc., Cary, NC) and Stata® version 11 (Stata Corp, College Station, TX) for statistical analyses.

### Regulatory approvals

The study was approved by the Institutional Review Board at VA Pittsburgh Healthcare System. Appropriate approvals were obtained from each of the databases from where data were retrieved.

## Results

### Baseline characteristics

**Fig. 1:**
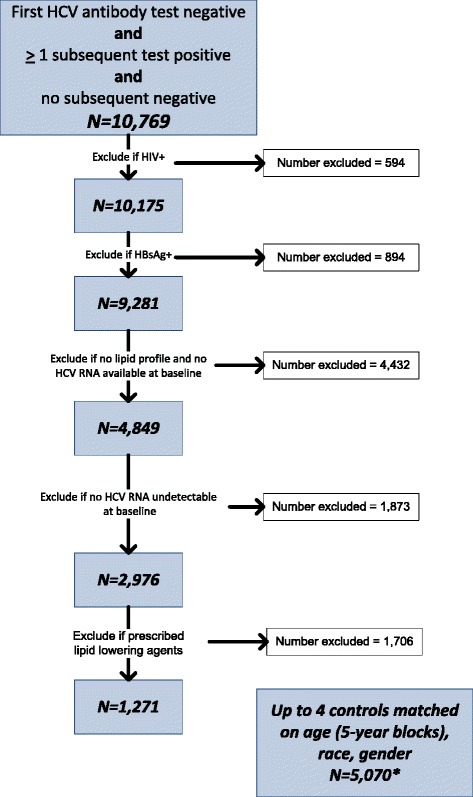
Study flow sheet

Within ERCHIVES we identified a total of 10,769 persons with a known window for HCV seroconversion (Fig. 1). After excluding those with HIV infection (*N* = 594), positive hepatitis B surface antigen (*N* = 894), missing lipid profiles or HCV RNA at baseline (*N* = 4,432) and those with undetectable HCV RNA at baseline (*N* = 1,873), there were 1,270 persons available for analysis. We identified 5,070 HCV uninfected controls matched on the criteria described above. Median age [IQR] was 47 [37,53] for HCV+ and 52 [47,57] for the HCV- group. There were 69 % White, 20 % Black and 3 % Hispanics and 91 % were male in each group. (Table 1) Mean BMI [SD] was 26.94 [6.73] in the HCV+ and 28.15 [5.98] in the HCV- group (*P < 0.001)*. The prevalence of hypertension was lower while alcohol and drug abuse and dependence diagnoses were higher among HCV+, compared with HCV- persons. Prevalence of stage 3–5 chronic kidney disease and cardiovascular disease was similar in both groups.

**Table 1:** Baseline characteristics

Variable	HCV+	Control*	*P*-value
	*N* = 1270	*N* = 5070	
Age, median (IQR)	47(37,53)	52(47,57)	<0.001
Race			0.99
White	69.21 %	69.31 %	
Black	20.00 %	19.90 %	
Hispanic	3.15 %	3.14 %	
Other/not known	7.64 %	7.65 %	
Gender (% male)	91.34 %	91.40 %	0.94
Body mass index, mean (kg/m^2^)	26.94(6.73)	28.15(5.98)	<0.001
Underweight (BMI <18.5)	1.02 %	2.37 %	0.003
Normal weight (BMI 18.5–24.9)	37.48 %	29.29 %	<0.001
Overweight (BMI 25–29.9)	39.69 %	36.21 %	0.02
Obese (BMI > =30)	21.81 %	32.13 %	<0.001
Diabetes	4.41 %	4.73 %	0.62
Hypertension	31.65 %	38.78 %	<0.001
Chronic kidney disease	13.78 %	15.50 %	0.13
Cardiovascular disease	3.39 %	4.18 %	0.2
Alcohol abuse or dependence	45.75 %	30.69 %	<0.001
Drug abuse or dependence	49.37 %	21.18 %	<0.001
ALT, mean (SD)	79.31(114.08)	33.28(22.48)	<0.001
AST, mean (SD)	56.36(67.91)	30.18(20.87)	<0.001
FIB-4, mean (SD)	1.39(1.7)	1.28(1.05)	0.03
Cirrhosis by FIB-4 > 3.5 (%)	4.86 %	2.95 %	<0.001
Total cholesterol, median (IQR)	178.5(154.5,199.5)	180(159,200)	0.02
> = 240 (%)	3.31 %	3.41 %	0.85
LDL, median (IQR)	103.5(83.7,122.5)	106(87.6,124.5)	0.003
> = 190 (%)	0.24 %	0.32 %	0.5
TG, median (IQR)	120(84,173)	110.75(77.5,159)	<0.001
> = 500 (%)	0.31 %	0.45 %	0.5
HDL, median (IQR)	45.5(37.4,55)	45.5(38,56.5)	0.04
<40 (%)	32.20 %	30.18 %	0.16
HCV RNA log 10 IU/ml, mean (SD)	4.28(2.81)	--	--
HCV genotype		N/A	
1	37.40 %		
2	5.04 %		
3	6.69 %		
4	0.31 %		
Not available	50.55 %		

*Matched samples

*TC* total cholesterol, *LDL* low density lipoproteins, *HDL* high density lipoprotein, *TG* triglycerides, *non-HDL-C* non-HDL cholesterol

There were small but statistically significant differences in all lipid fractions between HCV+ and HCV- groups. (Table 1) Total cholesterol, LDL and non-HDL-C were lower while TG and HDL was higher among HCV infected compared with HCV uninfected persons.

### Changes in lipid profile over time

**Fig. 2:**
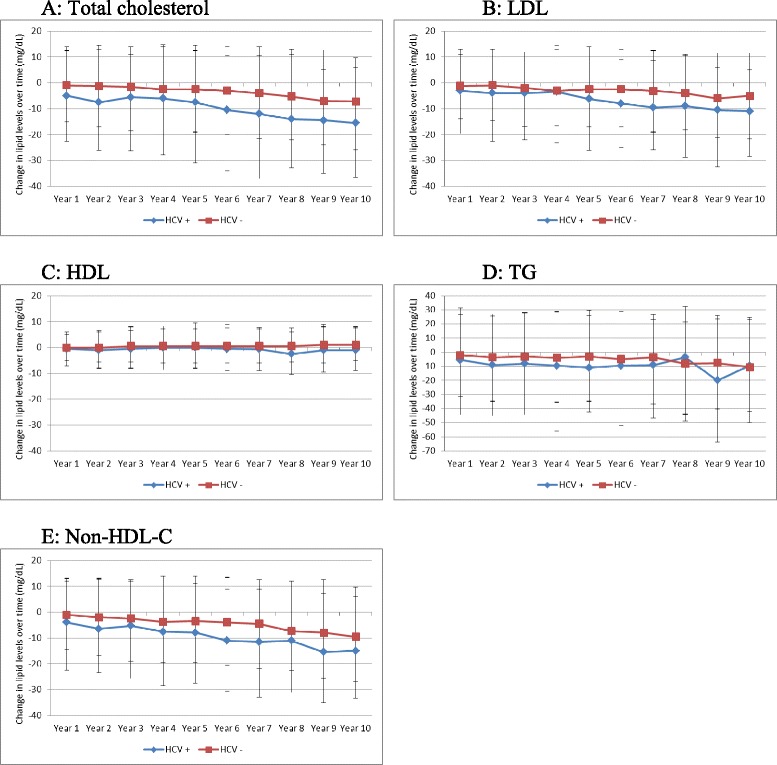
Changes in lipid levels over time among HCV seroconverted and HCV uninfected persons. **a** Changes in total cholesterol; **b** Changes in LDL-cholesterol; **c** Changes in HDL-cholesterol; **d** Changes in triglycerides; **e** Changes in non-HDL cholesterol

**Fig. 3:**
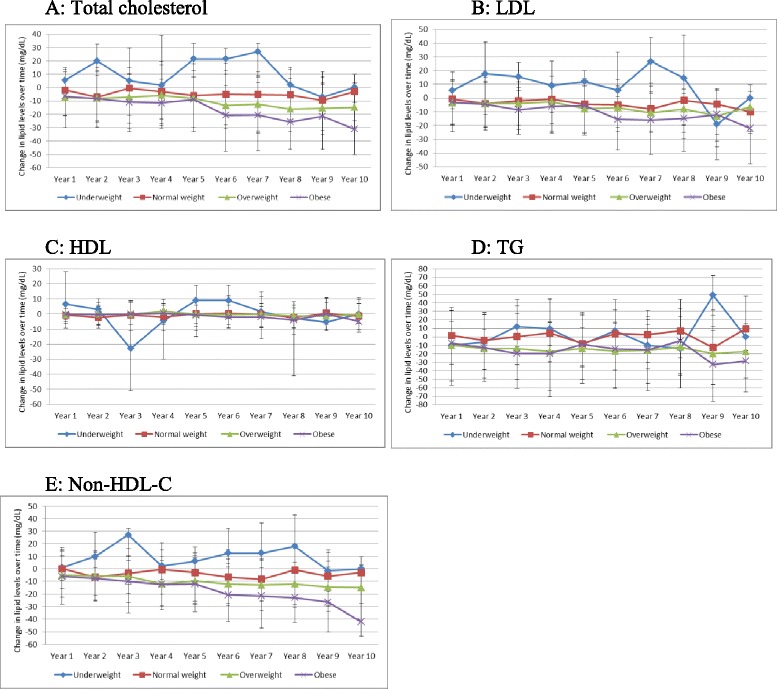
Changes in lipid levels over time among HCV seroconverted and HCV uninfected persons by BMI categories. **a** Changes in total cholesterol; **b** Changes in LDL-cholesterol; **c** Changes in HDL-cholesterol; **d** Changes in triglycerides; **e** Changes in non-HDL cholesterol

**Fig. 4:**
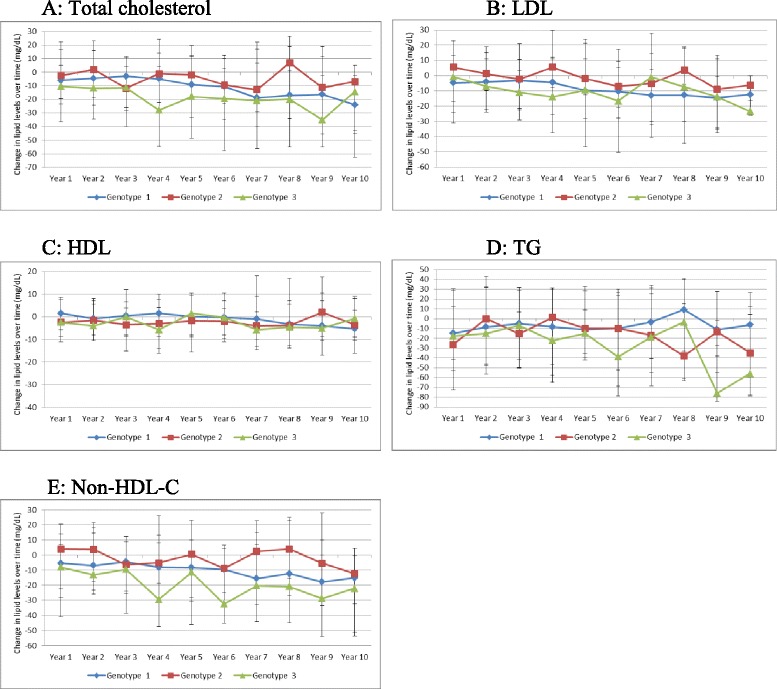
Changes in lipid levels among HCV seroconverted persons with available genotype results, by HCV genotype. **a** Changes in total cholesterol; **b** Changes in LDL-cholesterol; **c** Changes in HDL-cholesterol; **d** Changes in triglycerides; **e** Changes in non-HDL cholesterol

Over the period of follow up, there were significant changes in most lipid fractions. All non-HDL lipid fractions declined over time for both HCV+ and HCV- persons, with much larger declines observed in HCV+ compared with HCV- persons (Fig. 2). There was no consistent pattern of change in lipid fractions by BMI categories (Fig. 3). Among HCV+ with available genotype results, declines in lipid fractions were generally larger among genotype 3 infected persons, lowest in those with genotype 2 and intermediate in those with genotype 1 infected persons (Fig. 4). Among HCV+ persons, after adjusting for time-updated BMI and FIB-4 score, TC cholesterol decreased by (mean [SD] mg/dL) 12.06 [36.95], LDL decreased by 9.22 [31.44], TG decreased by 13.58[87.01] and non-HDL-C decreased by 12.55 [35.14]. Among HCV- persons, TC cholesterol decreased by (mean [SD] mg/dL) 4.15[31.21], LDL decreased by 4.16 [26.51], TG decreased by 4.42 [82.34] and non-HDL-C decreased by 5.78 [30.17] (*P-*value <0.001 for all comparisons with HCV+). (Table 2) Since BMI has a significant effect on several metabolic parameters, we plotted BMI for HCV+ and HCV- infected groups over time. (Additional file 1: Figure S1) The BMI in each group remained relatively stable over time and the difference between the groups that was observed at baseline appeared to remain constant. We plotted changed in lipid fractions over time in HCV+ and HCV- groups adjusted for time-updated FIB-4 score, and the results were similar to the unadjusted analysis. (Additional file 2: Figure S2) We also studied the impact of HCV RNA upon lipid levels. Within the HCV+ group, we divided subjects into tertiles based on HCV RNA levels at baseline and plotted lipid levels over time. (Additional file 3: Figure S3) No major differences were observed by HCV RNA levels.

**Table 2:** Changes in lipid levels by HCV status over time, adjusted for time-updated body mass index, and time-updated FIB-4 score (change from baseline to last observation)

	HCV+	HCV-	*P*-value
TC, mean(SD), mg/dL	−12.06(36.95)	−4.15(31.21)	<0.001
LDL, mean(SD), mg/dL	−9.22(31.44)	−4.16(26.51)	<0.001
HDL, mean(SD), mg/dL	0.58(17.04)	1.62(15.78)	<0.001
TG, mean(SD), mg/dL	−13.58(87.01)	−4.42(82.34)	<0.001
Non-HDL-C, mean(SD), mg/dL	−12.55(35.14)	−5.78(30.17)	<0.001

*TC* total cholesterol, *LDL* low density lipoproteins, *HDL* high density lipoprotein, *TG* triglycerides, *non-HDL-C* non-HDL cholesterol

## Discussion

In this study we observed progressive decline in lipid fractions other than HDL after HCV seroconversion. These declines were significantly more than in a demographically comparable HCV uninfected group, and remained significant after adjusting for BMI and degree of liver fibrosis. Within the HCV seroconverted group, the changes were generally larger in HCV genotype 3 infected persons, but were not related to the degree of HCV viremia.

Mounting evidence demonstrates a unique link between HCV and host lipid dysregulation. Patients infected with HCV have been shown to have significantly reduced levels of LDL and TC, compared to healthy matched controls and patients infected with hepatitis B. [19–22] HCV infection has also been associated with dynamic changes in circulating lipid levels. In a recent retrospective analysis of 38 patients with acute HCV comparing pre- and post-infection lipid levels, those with acute HCV showed a significant reduction in TC and LDL levels compared to baseline. [23] In addition, the observed hypolipidemia resolved with HCV viral eradication; those with spontaneous or treatment-induced viral clearance had a rebound in LDL and TC to levels at or above their pre-infection baseline. [23] Such dynamic changes in lipid levels suggest a direct interaction between the virus itself and host lipid metabolism. Due to lack of knowledge of precise time or duration of infection and/or lack of an appropriate control group, previous studies do not inform us whether the changes in lipid levels are a direct consequence of HCV infection, or such changes are present at time of HCV infection or even prior to that. Our study adds significantly to the current understanding by demonstrating that such changes occur predominantly after HCV seroconversion, indirect implicating a role of HCV infection in such changes. The lipid levels (TC, LDL, TG, non-HDL-C) were only marginally lower among the HCV infected group at baseline, and declined significantly more over time for HCV seroconverted persons compared with HCV uninfected controls. Differences in BMI alone do not fully explain this difference, since BMI remained relatively constant over time for both HCV+ and HCV- groups. While the timing suggests at least a partial role of HCV infection in such declines, these declines do not appear related to the degree of HCV viremia.

There is biological plausibility for our findings. HCV may alter cholesterol homeostasis through several mechanisms. HCV infection may produce hypolipidemia through its interference with the mevalonate pathway, resulting in decreased cholesterol synthesis. This diminished synthesis may then subsequently upregulate LDL-receptor expression, ultimately lowering LDL levels. [19, 23, 24] Hepatitis C viral proteins have been shown to directly activate the PI3-K/AKT signaling pathway, resulting in activation of the master regulator sterol response element binding protein (SREBP), which plays a critical role in activation of genes essential to fatty acid and cholesterol biosynthesis. [25–28] Additionally, infection with HCV is associated with reduced microsomal triglyceride transfer protein (MTTP), an enzyme critical for VLDL synthesis, and whose inhibition results in decreased circulating LDL and cholesterol levels. [29] We have previously demonstrated progressively increasing liver fibrosis among both HCV infected and uninfected Veterans, although the rate of progression and proportion of persons developing liver cirrhosis is much higher among HCV infected Veterans. [9] The aging population, particularly the Veterans in the US, have a high burden of comorbidities. High burden of comorbid illnesses, e.g. psychiatric disorders, severs CVD, cancer, etc., are associated with poor nutritional status, which in turn may lead to decreases in lipid levels.

The association of metabolic abnormalities and lipid profiles with various HCV genotypes is unknown. Infection with HCV genotype 3 is associated with a higher risk of hepatic steatosis, cirrhosis and hepatocellular carcinoma, but its association with insulin resistance has not been proven. [30–33] We observed a trend towards lower TC, LDL and non-HDL-C levels in HCV genotype 3 infected persons compared with HCV genotype 1. The significance of this is unclear and limited by the fact that genotype results were available for less than half of HCV infected persons.

Certain limitations of our study need to be considered when interpreting the results. While the window of seroconversion was quite clear, the window was wide and the precise time of infection could not be determined. Lipid measurements were done as part of routine clinical care and the fasting status of subjects was not known. Some lipid fraction measurements are more affected by measurement in a non-fasting state than others. The large number of subjects and multiple measurements for each subject over time is likely to have attenuated that effect.

## Conclusions

In conclusion, all lipid fractions other than HDL decline after seroconversion for HCV. Such declines are more pronounced than in comparable HCV uninfected persons and persist after adjusting for BMI and degree of liver fibrosis. Further studies are needed to determine if these differences in lipid levels translate into risk of cardiovascular disease and other clinical events, and to determine the need and optimal time for lipid lowering therapy in HCV infected persons.

## Additional files

Additional file 1:
**Figure S1.** Changes in body mass index over time in HCV+ and HCV- persons.(DOC 49 kb) Additional file 2:
**Figure S2.** Changes in lipid levels over time among HCV seroconverted and HCV uninfected, adjusted for time-updated FIB-4 score. (A) Changes in total cholesterol; (B) Changes in LDL-cholesterol; (C) Changes in HDL-cholesterol; (D) Changes in triglycerides; (E) Changes in non-HDL cholesterol.(DOC 109 kb) Additional file 3:
**Figure S3.** Changes in lipid levels among HCV seroconverted persons, by HCV RNA tertiles. (A) Changes in total cholesterol; (B) Changes in LDL-cholesterol; (C) Changes in HDL-cholesterol; (D) Changes in triglycerides; (E) Changes in non-HDL cholesterol.(DOC 126 kb)
