# Supplementary figures and images for: Changes in circulating lipids level over time after acquiring HCV infection: results from ERCHIVES
Source: BMC Infect Dis. 2015 Nov 11;15:510. doi: 10.1186/s12879-015-1268-2 (PMC4642733; doi:10.1186/s12879-015-1268-2)

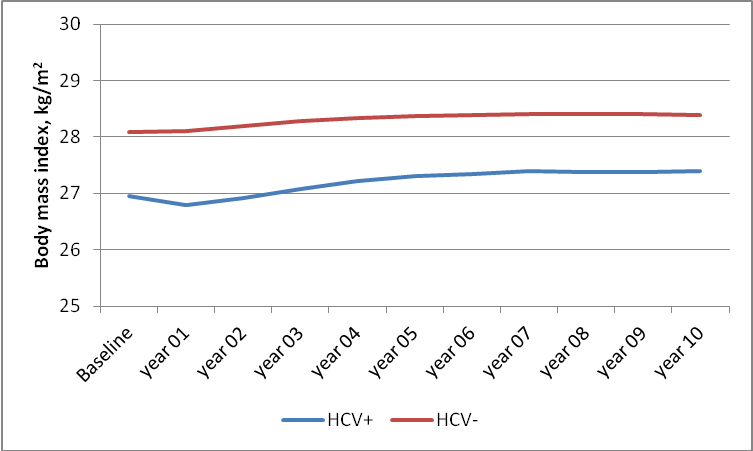


**Supplementary figure 1. Changes in body mass index over time in HCV+ and HCV- persons.**

Supplement: Additional file 1: — Figure S1. Changes in body mass index over time in HCV+ and HCV- persons.(DOC 49 kb) [file 12879_2015_1268_MOESM1_ESM.doc]
