# Supplementary material for: Changes in circulating lipids level over time after acquiring HCV infection: results from ERCHIVES
Source: BMC Infect Dis. 2015 Nov 11;15:510. doi: 10.1186/s12879-015-1268-2 (PMC4642733; doi:10.1186/s12879-015-1268-2)
Supplement: Additional file 3: — Figure S3. Changes in lipid levels among HCV seroconverted persons, by HCV RNA tertiles. (A) Changes in total cholesterol; (B) Changes in LDL-cholesterol; (C) Changes in HDL-cholesterol; (D) Changes in triglycerides; (E) Changes in non-HDL cholesterol.(DOC 126 kb) [file 12879_2015_1268_MOESM3_ESM.doc]

Panel A: Total cholesterol Panel B: LDL


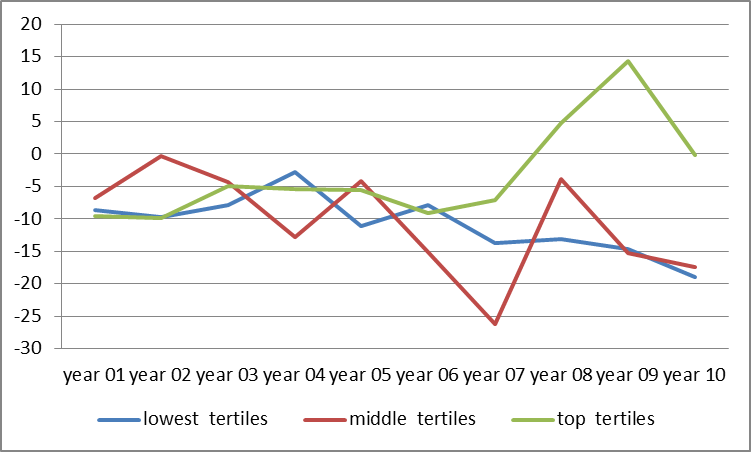

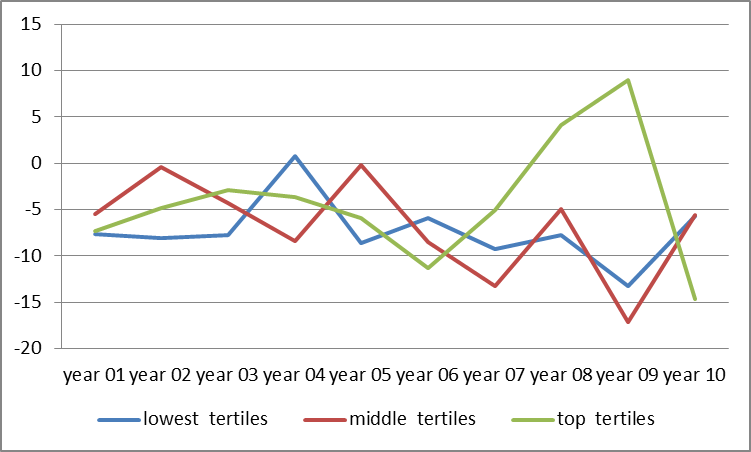


Panel C: HDL Panel D: TG


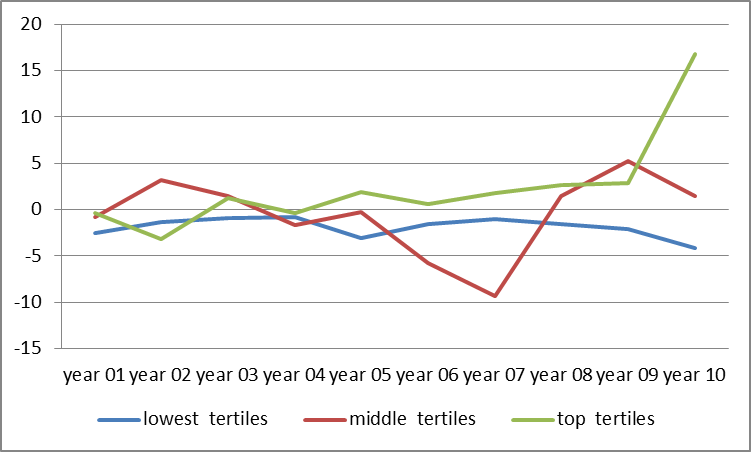

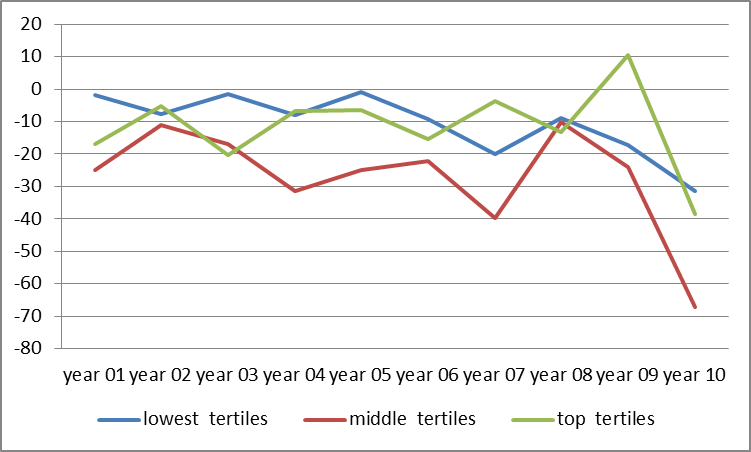


Panel E: Non-HDL-C


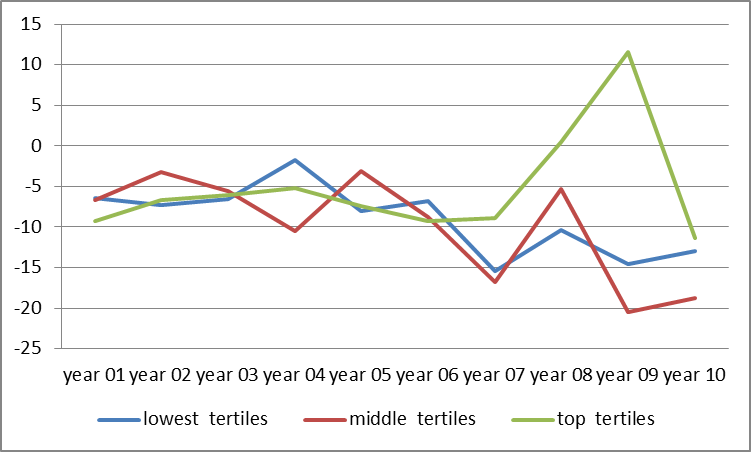


**Supplementary figure 3. Changes in lipid levels among HCV seroconverted persons, by HCV RNA tertiles.**

(A) Changes in total cholesterol; (B) Changes in LDL-cholesterol; (C) Changes in HDL-cholesterol; (D) Changes in triglycerides; (E) Changes in non-HDL cholesterol
